# L-carnitine co-administration prevents colistin-induced mitochondrial permeability transition and reduces the risk of acute kidney injury in mice

**DOI:** 10.1038/s41598-024-67171-x

**Published:** 2024-07-16

**Authors:** Sophia L. Samodelov, Zhibo Gai, Francesca De Luca, Klara Haldimann, Sven N. Hobbie, Daniel Müller, Gerd A. Kullak-Ublick, Michele Visentin

**Affiliations:** 1 Department of Clinical Pharmacology and Toxicology, University Hospital Zürich, University of Zürich, 8006 Zürich, Switzerland; 2 Experimental Center, Shandong University of Traditional Chinese Medicine, Jinan, 250355 China; 3 Institute of Medical Microbiology, University of Zürich, 8006 Zürich, Switzerland; 4 Institute of Clinical Chemistry, University Hospital Zürich, University of Zürich, 8006 Zürich, Switzerland; 5 Mechanistic Safety, Patient Safety & Pharmacovigilance, Clinical Development and Medical Affairs, Novartis Pharma, 4056 Basel, Switzerland; 6 Present Address: Laboratory Medicine, University of Basel, 4056 Basel, Switzerland

**Keywords:** Colistin, Drug-induced kidney injury, Kim-1, L-carnitine, Mitochondria, Nephrotoxicity polymyxins, Proximal tubule, Medical research, Molecular medicine, Nephrology, Kidney diseases

## Abstract

Colistin is a polymyxin antibiotic currently experiencing renewed clinical interest due to its efficacy in the treatment of multidrug resistant (MDR) bacterial infections. The frequent onset of acute dose-dependent kidney injury, with the potential of leading to long-term renal damage, has limited its use and hampered adequate dosing regimens, increasing the risk of suboptimal plasma concentrations during treatment. The mechanism of colistin-induced renal toxicity has been postulated to stem from mitochondrial damage, yet there is no direct evidence of colistin acting as a mitochondrial toxin. The aim of this study was to evaluate whether colistin can directly induce mitochondrial toxicity and, if so, uncover the underlying molecular mechanism. We found that colistin leads to a rapid permeability transition of mitochondria isolated from mouse kidney that was fully prevented by co-incubation of the mitochondria with desensitizers of the mitochondrial transition pore cyclosporin A or L-carnitine. The protective effect of L-carnitine was confirmed in experiments in primary cultured mouse tubular cells. Consistently, the relative risk of colistin-induced kidney damage, calculated based on histological analysis as well as by the early marker of tubular kidney injury, Kim-1, was halved under co-administration with L-carnitine in vivo. Notably, L-carnitine neither affected the pharmacokinetics of colistin nor its antimicrobial activity against relevant bacterial strains. In conclusion, colistin targets the mitochondria and induces permeability transition thereof. L-carnitine prevents colistin-induced permeability transition in vitro. Moreover, L-carnitine co-administration confers partial nephroprotection in mice treated with colistin, without interfering with its pharmacokinetics and antibacterial activity.

## Introduction

Colistin is a polymyxin antibiotic that has been available for clinical use since the 1960s, but was replaced shortly thereafter with supposedly more tolerable antibiotics. In the last ten years, colistin has experienced a renewed clinical interest as a last resort for the treatment of multidrug-resistant bacteria such as methicillin-resistant *Staphylococcus aureus*, vancomycin-resistant *enterococci* and some Gram-negative bacilli like *Pseudomonas aeruginosa*, *Acinetobacter baumannii* and *Enterobacterales*^1,2^. Polymyxins are antimicrobial peptides that interact with the lipopolysaccharide (LPS) moiety of the membrane of Gram-negative bacteria and induce divalent cation displacement, resulting in destabilization of the membrane, loss of integrity, and cell death^3–5^. Colistin’s efficacy depends upon achieving adequate levels in the blood of the patients, whereas dosing regimens have been largely empirically determined in the past decades of revisited use. Modern pharmacokinetics has underscored the importance of a loading dose to achieve circulating values above bacterial Minimal Inhibitory Concentrations (MIC) to reduce the risk of resistance^6,7^. However, the use of a colistin-loading dose is hampered by the rather early onset of acute kidney injury (AKI). Because of the lack of harmonized criteria to define AKI, the incidence of colistin-induced nephrotoxicity varies among studies^8–11^. However, when KDIGO guidelines are retrospectively applied, 73 out of 249 patients receiving colistin developed AKI at 7 days after treatment initiation. Even more alarming is that 23% of the AKI cases (17/73) required renal replacement therapy^12^.

The mechanism underlying colistin-induced nephrotoxicity is not fully understood, but it arguably initiates with extensive active reabsorption and accumulation of the drug in proximal tubular cells. Candidate transporters and receptors involved in colistin uptake across the brush-border membrane of the proximal tubular cells are the Glycosylphosphatidylinositol (GPI)-anchored protein megalin^13^, the human peptide transporter 2 (PEPT2, *SLC15A2*) and the carnitine/organic cation transporter 2 (OCTN2, *SLC22A5*)^14,15^. All three proteins are highly expressed at the luminal side of proximal tubular cells^16–19^. Studies in mice showed that colistin toxicity is associated with the activation of (i) the mitochondrial apoptotic pathway, (ii) the death receptor pathway, and (iii) the endoplasmic reticulum pathway^20,21^. Yet, the primary target of colistin in eukaryotic cells has not been identified. Experiments with *Acholeplasma laidlawii B* bacteria showed that the depletion of cardiolipin confers resistance to polymyxins^22^. Furthermore, polymyxins have been shown to inhibit cellular respiration in Gram-negative and Gram-positive bacteria by inhibiting the NADH-Q oxidoreductase (NDH-2) of complex I^23–25^. Taken together, these data suggest that once inside the cells, colistin might directly attack mitochondria. In the present study, we demonstrate that freshly isolated mitochondria rapidly depolarize upon exposure to colistin and that pharmacological inhibition of the opening of the mitochondrial permeability transition pore (mPTP) protected proximal tubular cells from colistin-induced cytotoxicity in vitro and reduced the risk of kidney injury in mice.

## Methods

### Drug solutions

Colistin sulfate salt (*hereinafter* “colistin”) with a specific activity ≥ 19,000 IU/mg (#C4461) was purchased from Sigma Aldrich (St. Louis, MO, USA). For in vitro experiments, colistin was dissolved in dH_2_O, aliquoted, and kept at −20 °C. The solution was discarded after approximately one month from the day of the preparation. L-Carnitine hydrochloride (*hereinafter* “L-carnitine”) (#C0283) was purchased from Sigma Aldrich (St. Louis, MO, USA). L-Carnitine was dissolved in dH_2_O, aliquoted, and kept at −20 °C. For in vivo experiments, colistin and L-carnitine were dissolved in PBS on each treatment day prior to injection.

### Mouse husbandry

For the isolation of mitochondria and proximal tubular cells, kidneys were excised from cadavers of 8–12-week-old male and female wild type C57/BJ and C57/BJ crossed with 129S6 used for other experimental purposes not related to this study, following the principles of the 3Rs (Replacement, Reduction and Refinement) of animal experimentation. For in vivo experiments, 10–12-week-old female C57/BJ mice (Charles River Laboratories, Wilmington, MA, USA) were purchased and acclimatized for 2 weeks before the initiation of the experiments. Additionally, mock procedure to acclimatize mice to handling for *i.p.* injection and weighing was carried out on the two consecutive days before beginning the experiments. At the end of the experiments, mice were euthanized via CO_2_ inhalation. Animal experiments and protocols conformed to the Guide for the Care and Use of Laboratory Animals (US National Institutes of Health), the Swiss animal protection laws and were approved by the Cantonal Veterinary Office in Zurich, Switzerland (study number 186/2017).

### Primary culture of mouse tubular cells

Primary tubular cells were isolated according to a method previously described^26,27^. Kidney cortices were dissected, sliced, minced, and shaken at 37 °C for 1 h in a 0.25% trypsin/EDTA solution. Then, the trypsin/EDTA solution was neutralized with Dulbecco’s modified Eagle’s medium (DMEM, #22320-022, ThermoFisher Scientific, Waltham, MA, USA), supplemented with 10% (v/v) fetal bovine serum (FBS, #S181C, Biowest, Nuaillé, France). The cell suspension was passed through a 100-μm cell strainer and then centrifuged at 72.4*g*_*av*_ for 5 min at room temperature. The pellet containing the tubular cells was washed with 10 mL of DMEM/FBS and then centrifuged at 72.4*g*_*av*_ for 5 min at room temperature. The washing step was repeated and then the pellet, mostly consisting of tubular cells, was resuspended in Complete Renal Epithelial Cell Growth Medium (REGM, #CC-3190, Lonza, Basel, Switzerland). Cells were cultured at 37 °C in a humidified atmosphere of 5% CO_2_ with medium changed every 2 days until confluence.

### Mitochondria isolation

Functional mitochondria were isolated following the method described in^28^. Briefly, after excision, the kidneys were placed in ice-cold isolation buffer (200 mM mannitol, 50 mM sucrose, 5 mM KH_2_PO_4_, 5 mM MOPS, 0.1% fatty acid free BSA, 1 mM EGTA, adjusted to pH 7.15 with KOH), homogenized with a motor-driven tightly fitting glass-Teflon Potter grinder operated at 1,600 rpm and centrifuged at 4 °C for 3 min, at 1,100*g*_*av*_. The supernatant was centrifuged at 4 °C for 10 min at 10,000*g*_*av*_. The pellet containing the mitochondrial fraction was resuspended in incubation buffer (120 mM KCl, 10 mM Tris, 5 mM KH_2_PO_4_, adjusted to pH 7.4 with HCl). For oxygen consumption measurement, the mitochondria were resuspended in MiR05 medium (0.5 mM EGTA, 3 mM MgCl_2_, 60 mM lactobionic acid, 20 mM taurine, 10 mM KH_2_PO_4_, 20 mM HEPES, 110 mM Sucrose, 0.1% BSA, adjusted to pH 7.1 with KOH). Mitochondria were kept on ice and used within four hours upon completion of the isolation.

### Assessment of mitochondrial membrane potential

Membrane potential was assessed using rhodamine 123 (Rho123, #83702, Sigma-Aldrich, St. Louis, MO) (λ_ex_ = 488 nm, λ_em_ = 527 nm), a fluorescent dye, whose uptake into mitochondria is driven by the membrane potential across the inner membrane. For assessment of mitochondrial membrane potential in cultured cells, primary cultured tubular cells were seeded at the density of 5 × 10^4^ cells/well on an 8-well open µ-slide chambered coverslip. Once reaching near-confluence, culture medium was aspirated and cells were washed once with PBS, followed by a 20 min incubation with NucBlue™ Live ReadyProbes™ Reagent (#R37605, ThermoFisher Scientific, Waltham, MA, USA) to stain the nuclei. Afterwards, cells were washed twice with PBS, and then incubated for 20 min at room temperature with Rho123 diluted in respiration buffer (130 mM KCl, 5 mM K_2_HPO_4_, 20 mM MOPS, 2.5 mM EGTA, 3 mM succinate, 0.1% BSA, adjusted to pH 7.15 with KOH) to a final concentration of 1 µM. Finally, cells were washed twice with PBS before confocal microscopy (DMI6000B, Leica Microsystems, Wetzlar, Germany). The mitochondrial membrane potential in isolated mitochondria was assessed in quenching-mode, that is, when the level of Rho123 in the mitochondria is sufficiently high, its emission wavelength shifts towards the red, resulting in a reduction of the fluorescent signal at 527 nm^29^. To this end, Rho123 was dissolved in respiration buffer at the final concentration of 10 µM. Then, the fluorescent signal was recorded at 527 nm in the absence of mitochondria (maximal signal) and monitored over time after adding the mitochondria suspension, until the equilibrium was reached (maximal quenching).

### Oxygen consumption rate (OCR) measurement

Oxygen consumption was assessed in the O2k-FluorRespirometer (Oroboros Instruments GmbH, Innsbruck, Austria). A pre-calibrated 2-mL chamber was filled with MiR05 and pre-warmed at 37 °C. A mini-stirrer was inserted in the chamber and the stirring speed set at 750 rpm. The effect of colistin and L-carnitine on OCR was assessed in mitochondria in state II. An aliquot from the mitochondria suspension was injected in the chamber and the basal OCR was recorded (state I). Then, mitochondria were put into state II by adding 5 mM succinate. Data were acquired in real-time using the DatLab 7 software. OCR values were corrected for the O_2_ background value (noise) and expressed as pmol of O_2_·sec^-1^·ml^-1^. For each titration, the average value from a one-minute interval at stable OCR was used for data analysis. See the supplementary information for details on delta OCR data analysis and interpretation in these experiments.

### Immunofluorescence

Primary cultured tubular cells were seeded at the density of 5 × 10^4^ cells/well on an 8-well open µ-slide chambered coverslip. Confluent cell cultures were washed once with PBS, and then incubated for 20 min at room temperature with NucBlue™ Live ReadyProbes™ Reagent (#R37605, ThermoFisher Scientific, Waltham, MA, USA) to stain the nuclei, washed with PBS, permeabilized with 0.1% Triton-X 100 for 30 min, fixed at 37 °C for 20 min with 4% Paraformaldehyde in PBS, and blocked with 2% BSA in PBS at room temperature for 20 min. Cells were then probed overnight at 4 °C for GRP78 BiP (1:500, #ab21685, Abcam, Cambridge, UK). Afterwards, cells were washed with PBS, and incubated at room temperature for 30 min with an anti-rabbit IgG Alexa Fluor 546 antibody (1:100, #A-11035, Thermo Fisher Scientific, Waltham, MA, USA). Cells were washed with PBS before image acquisition by confocal microscopy (DMI6000B, Leica Microsystems, Wetzlar, Germany).

### Determination of minimal inhibitory concentration (MIC)

Determination of MIC was assessed by a standard broth microdilution (BMD) assay according to the Clinical Laboratory and Standards Institute^30^, in cation-adjusted Mueller–Hinton broth (CAMHB). Bacterial strains comprised the CLSI quality control strains *E. coli* ATCC 25922 and *P. aeruginosa* ATCC 27853, as well as *A.* *baumannii* NCTC 13304, *K. pneumoniae* ATCC 700603, and *E. coli* strain NCTC 13846 with an MCR-1-mediated colistin resistance. Changes in susceptibility of > 1 log_2_ titer were considered as significant deviations from the control group.

### Animal studies

For the nephrotoxicity study, mice were randomly housed (as shipped) in four individually ventilated cages (2–5 mice/cage) with free access to food and water. Three separate experiments were performed, once with 2–3 mice per group due to logistical issues, and twice with 5 mice, for a total of 12–13 mice per treatment group as follows: vehicle (PBS) n = 12, L-carnitine alone n = 13, colistin alone n = 13, colistin and L-carnitine co-injection = n = 12. Mice were *i.p.* injected daily for seven days. Spontaneous urination samples were collected during handling prior to injections on each treatment day and before euthanization. No urine sample via spontaneous urination or by gently stroking the lower abdomen could be obtained immediately prior to euthanization for a total of 3 mice; 2 from the L-carnitine treatment group and 1 from the colistin treatment group. In addition, urinary creatinine could not be detected or was abnormally low in the final urine sample for two mice from the colistin + L-carnitine treatment group. For these technical reasons, urinary Kim-1 values are reported for n = 12 vehicle (PBS), n = 11 L-carnitine alone, n = 12 colistin alone, and n = 10 colistin and L-carnitine co-injection (Table 3 Relative Risk, (Fig. 4). Animals were sacrificed and kidneys were fixed or snap frozen and kept at −80 °C. For the pharmacokinetic study, mice were randomly separated in two individually ventilated cages (4 mice/cage) with free access to food and water. Mice were subjected to single *i.p.* injection and tail-tip-bleeding was performed from each animal at 20, 40, 60, 120, and 240 min after injection, sampling 20–25 μL of blood per time point as illustrated in (Fig. 3A). Samples obtained from the same time point and same treatment group were pooled in BD Microtainer SST Tubes (#365968, Becton, Dickson and Company, Franklin Lakes, New Jersey, USA), spun down, and the serum stored at −80 °C for colistin composite pharmacokinetics. Upon collection of the last sample (i.e., 240 min after injection), the animal was sacrificed via CO_2_ inhalation, and the kidneys excised, snap frozen and kept at −80 °C until colistin quantification.

### Histology

Upon longitudinal dissection of the right kidney, one half was incubated at 4 °C overnight with 4% (w/v) paraformaldehyde (PFA) in PBS for histological examination on 5-μm sections. Microwave-based antigen retrieval with citrate buffer (#S2369, Agilent Technologies, Santa Clara, CA, USA) was followed by incubation with antibodies against Kim-1 (1:250, #NBP1-76701, Novus Biologicals, Centennial, CO, USA) or Ngal (1:200, #ab63929, Abcam, Cambridge, UK). Detection was performed using the Dako EnVision™ + Dual Link System-HRP DAB + (#K4065, Dako/Agilent Technologies, Santa Clara, CA, USA). After counterstaining performed with Mayer’s hematoxylin solution (#3870, Biosystems Switzerland AG, Muttenz, CH), the sections were dehydrated, and mounted using Organo/Limonene Mount (#O8015, Sigma Aldrich, St. Louis, MO). For hematoxylin/eosin Y staining (HE), sections were rehydrated and stained with Mayer’s hematoxylin and counterstained with Eosin Y solution (#318906, Sigma Aldrich, St. Louis, MO). All sections were imaged at 5x, 10x, and 20 × magnification, with 2 images per section per magnification being captured. For quantification of severity and staining scores, two experienced scientists carried out a histological evaluation of 4 images per mouse at 2 magnifications, independently and in a blinded manner. For the assessment of kidney injury severity (severity score), HE stained section images at 10 × and 20× (2 at each magnification, for a total of 4 images per mouse) were graded from 0 (no damage) to 5 (most severe damage observed across the samples analyzed), based on the following parameters: (i) protein cast formation, (ii) visible tubular dilation, (iii) loss of proximal tubular cell polarization (no visible brush-border membranes) (iv) area affected (with single nephron damage to majority of the cortex with visual signs of injury). For the quantification of Kim-1 and Ngal stainings (staining score), 5× and 10× images (2 at each magnification, for a total of 4 per mouse) were likewise graded with 0 (no staining), 1 (mild), 2 (moderate), or 3 (intense) based on intensity and area of the staining. Scores of each image across the scorers were similar, and averaged per image and per mouse.

### Detection of kidney injury markers in serum and urine

A colorimetric/fluorometric kit was used for the detection of creatinine (#K625, Biovision, Milpitas, CA, USA) in urine and serum. A commercially available ELISA test was used to measure Kim-1 (#ab213477, Abcam, Cambridge, UK) in urine. Urinary Kim-1 values were normalized for the urinary creatinine levels.

### Liquid chromatography and tandem mass spectrometry–colistin quantification in kidney and serum

One whole kidney was resuspended in ice-cold PBS and homogenized in a motor-driven tightly fitting glass-Teflon Potter grinder operated at 3,000 rpm and centrifuged at 4 °C for 20 min, at 2,000*g*_*av*_. The supernatant was collected and kept at −80 °C until the analysis. A 50-µl aliquot (serum or kidney homogenate) was diluted with 50 µl of aqueous solution containing the internal standard polymyxin B. After centrifugation, samples were measured by liquid chromatography coupled to tandem mass spectrometry (LC-MS/MS). Analysis was performed on a Sciex Qtrap 6500 triple quadrupole mass spectrometer (AB Sciex, Toronto, Canada) operated in positive heated electrospray ionization mode, controlled by Analyst 1.62. After online extraction with turbulent flow chromatography using a Cyclone 50 × 0.5 mm column (ThermoFisher Scientific, Waltham, MA, USA), analytes were separated on an Accucore C18 100 × 3 mm column (ThermoFisher Scientific, Waltham, MA, USA). As solvents, aqueous 0.5% formic acid (A), 0.5% formic acid in acetonitrile + 10% water (v/v) (B) and acetonitrile/2-propanol/acetone (1/1/1, v/v/v) (C) were used. After online extraction with 100% mobile phase A, analytes were transferred to the analytical column using 70% A and 30% B. On the analytical column, eluent composition was ramped from 99% A to 85% A in 1 min and to 70% A in 0.2 min with a flow rate of 0.45–0.5 ml/min. After holding the condition for 1.3 min, columns were cleaned up with 100% C and re-equilibrated to the initial conditions within 2 min. Protocol details can be found in Supplementary Table S1.

### Statistical analyses

Pharmacokinetic and statistical analyses were performed using GraphPad Prism Version 9. Statistical tests employed are reported in each figure legend and table footnote. For the calculation of relative risk, the open access MedCalc’s relative risk calculator was used (https://www.medcalc.org/calc/relative_risk.php).

### Ethics

Experiments involving animals are described in accordance with ARRIVE guidelines.

## Results

### Effect of colistin on mitochondrial membrane potential and oxygen consumption rate in freshly isolated mitochondria

**Figure 1:**
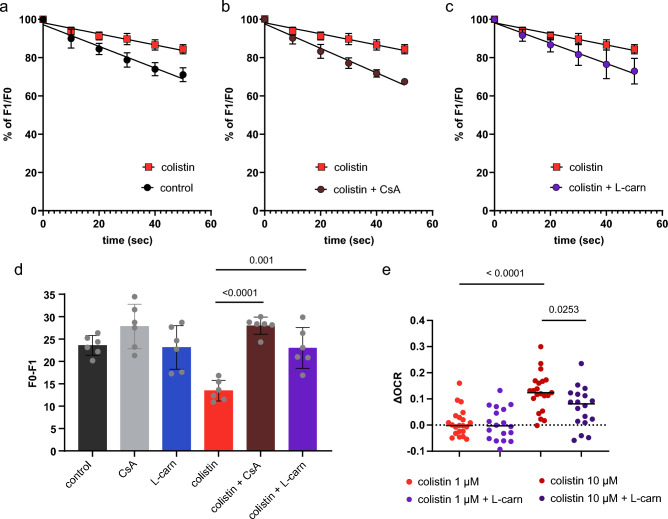
Mitochondrial membrane potential (MMP) and oxygen consumption rate (OCR) in isolated mitochondria. (**A**–**C**): Fluorescent Rho123 dye enters mitochondria driven by the mitochondrial membrane potential across the inner membrane. The accumulation of Rho123 in isolated mitochondria can be monitored by a decreased fluorescence emission at 527 nm. Mitochondria freshly isolated from mouse kidney were incubated for 1 h with water or 1 µM colistin (**A**), with 1 µM Cyclosporin A (**B**), or 250 µM L-carnitine (**C**), then mixed with Rho123 solution and the decline of the fluorescent signal was monitored over time. Data represent the mean ± SD from six independent experiments and expressed relative to the baseline fluorescent signal (F0) recorded in the absence of mitochondria. Slopes were compared by unpaired t-test. Additional control treatment groups are shown in supplementary Fig S2. (**D**) The net accumulation of Rho123 after 60-s incubation in mitochondria incubated for one hour with the indicated conditions prior to analysis. Data represent the mean ± SD from six independent experiments and expressed as difference between baseline (F0) and signal after one-minute incubation with Rho123 (F1). (**E**) The effect of colistin on OCR of mitochondria in State 2 (in the presence of succinate as a substrate) was monitored in the presence or absence of 250 μM L-carnitine. Each dataset was normalized for the respective State 2 OCR and expressed as the difference in the OCR measured in the untreated mitochondria (control). From one isolation, one to three measurements were performed. Data were generated from nine separate mitochondrial isolations. Statistically significant differences were calculated using one-way analysis of variance followed by Šidák’s post-hoc test. Additional data is shown in Fig. S1.

To assess the mitochondrial toxicity of colistin, freshly isolated mitochondria were incubated for one hour with 1 µM colistin, and then the quenching of the Rho123 fluorescent signal was monitored over time. In Fig. 1A, it can be seen that the reduction of the fluorescent signal was linear over time, reflecting the unidirectional flux of Rho123 into the mitochondria. The uptake rate of Rho123 was markedly lower in the mitochondria pre-treated with colistin than that in the untreated mitochondria (slope −0.29 vs −0.57, P = 0.0005), indicating that colistin induced the depolarization of the mitochondrial inner membrane. To determine whether the colistin-induced depolarization was the result of the opening of the mitochondrial permeability transition pore (mPTP), mitochondria were co-incubated with colistin and cyclosporin A, a well-characterized desensitizer of the mPTP^31,32^. Figure 1B shows that co-incubation with 1 µM cyclosporin A could prevent colistin-induced mPTP depolarization (slope −0.0029 vs −0.64, P < 0.0001). A similar effect was observed when cyclosporin A was replaced by the polyamine L-carnitine (slope −0.29 vs −0.53, P = 0.004) (Fig. 1C), which has been previously shown to desensitize the mPTP as well^33^. The effect of the above-mentioned combinations on the mitochondrial membrane potential was confirmed when comparing values at a later time point (60 s), when the Rho123 fluorescent signal deflected from linearity to approach the equilibrium (Fig. 1D). Notably, neither cyclosporin A nor L-carnitine could rescue the membrane depolarization induced by the proton uncoupler FCCP (not shown). Taken together, the data indicate that colistin-induced mitochondrial depolarization occurs upon the opening of the mPTP. Usually, mitochondrial depolarization is associated with an increase in the oxygen consumption. Figure 1E shows that the OCR of mitochondria exposed to colistin at the concentration of 1 μM was comparable to that of mitochondria exposed to an equal volume of vehicle (dH_2_O). However, OCR increased when the concentration of colistin was increased to 10 μM. Furthermore, it can be seen that the OCR elevation induced by 10 μM colistin was significantly reduced by the presence of L-carnitine.

### Effect of L-carnitine on colistin-induced mitochondrial membrane depolarization in primary cultured tubular cells

**Figure 2:**
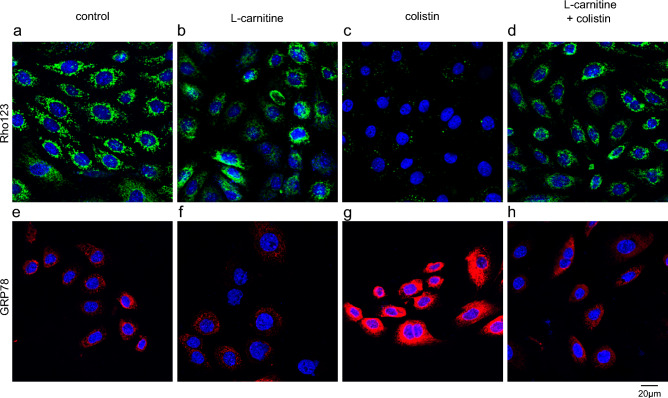
Mitochondrial membrane potential (MMP) and ER stress in primary cultured proximal tubular cells. Mitochondrial membrane potential (Rho123 staining, **A**–**D**) and ER stress (Grp78 staining, **E**–**H**) in primary cultured mouse proximal tubular cells after 24 h treatment with colistin at the extracellular concentration of 100 μM, in the presence or absence of L-carnitine at the extracellular concentration of 1 mM. Representative pictures from one experiment performed three times independently.

The protective effect of L-carnitine on colistin-induced mitochondrial toxicity was further investigated at the cellular level using primary cultured murine tubular cells. Cells were incubated for 24 h with colistin at the extracellular concentration of 100 µM in the presence or absence of 1 mM extracellular L-carnitine. Cells exposed to colistin were characterized by depolarized mitochondria. The co-incubation with L-carnitine fully protected the cells from colistin-induced mitochondrial depolarization (Fig. 2). To assess whether the level of ER stress, which is often coupled to mitochondrial dysfunction^34^, may be impacted by colistin treatment and rescued by co-incubation with L-carnitine, a staining against the ER stress marker GRP78/BiP was conducted. Expression of GRP78/BiP was elevated in the cells exposed to colistin, while not in those co-incubated with L-carnitine (Fig. 2).

### Effect of L-carnitine on the antimicrobial activity and pharmacokinetics of colistin

**Figure 3:**
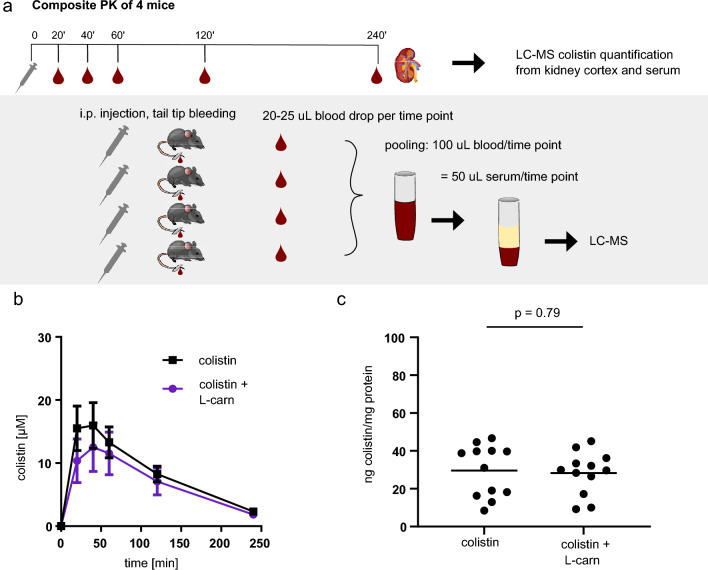
Pharmacokinetics and renal tissue accumulation of colistin. (**A**) Scheme of composite pharmacokinetic (PK) experimental design, using four mice per treatment group per experiment. Blood was collected by serial tail-tip sampling for each mouse, where 20–25 µL of blood were taken from each mouse per time point, and pooled per time point for analysis of colistin content in plasma. (**B**) Plasma colistin over time after a single *i.p*. dose of 20 mg/kg colistin with or without co-injection of 30 mg/kg L-carnitine. Points displayed are the average of at least three independent experiments using four mice per treatment group. (**C**) Colistin level in the renal tissue of the mice four hours after single *i.p*. dose of 20 mg/kg colistin with or without co-injection of 30 mg/kg L-carnitine. Whole kidneys were removed post-mortem and processed via homogenation and centrifugation before assessing colistin content. Each data point represents one mouse. P-values were calculated from one-way analysis of variance followed by Tukey’s post-hoc test.

Because L-carnitine is a unique carbon and nitrogen source, as well as an efficient osmoprotectant for bacteria^35–37^, we assessed the effect of L-carnitine on the antimicrobial activity of colistin in relevant bacterial strains. The colistin MIC in the presence of increasing concentrations of L-carnitine was determined for five bacterial strains; including the colistin-resistant *E. coli* NCTC 13846 strain (Table 1). L-carnitine at extracellular concentrations of 0.25–1 mM did not affect the antibacterial activity of colistin in the five strains tested. Deviations ≤ 1 log_2_ titer were considered to be within natural technical variation of the BMD assay. Additionally, no growth inhibition was observed with the positive control of 1 mM L-carnitine. Next, we assessed the effect of L-carnitine co-injection on colistin pharmacokinetics and accumulation in the kidney tissue in mice. The dose of colistin (20 mg/kg) was chosen based on our previous study, showing that all animals treated at this dose showed signs of acute kidney injury after 7 days of treatment^38^. The dose of L-carnitine (30 mg/kg) was chosen based on previous studies in rodents, and as well as on available pharmacokinetics and safety data generated in patients with primary carnitine deficiency^39,40^. Area under the curve (AUC) and C_max_ were not significantly affected by L-carnitine (Fig. 3B and Table 2). Likewise, the renal accumulation of colistin was comparable between the two groups (Fig. 3C).

**Table 1:** Antimicrobial activity of colistin.

MIC of Colistin (μg/mL, *n* = 3)					
	*E. coli*		*P. aeruginosa*	*A. baumannii*	*K. pneumoniae*
	WT	*mcr-1*	WT	WT	WT
L-carnitine (mM)	ATCC 25922	NCTC 13846	ATCC 27853	NCTC 13304	ATCC 700603
0	0.25	4	1	0.5–1	0.25
0.25	0.25	8	1	0.5	0.25
0.5	0.25	8–16	1	0.5	0.25–0.5
1	0.25	4	1	0.5	0.25

*MIC* minimal inhibitory concentration, *mcr-1* mobilized colistin resistant gene 1.

**Table 2:** Pharmacokinetic parameters of colistin.

Parameter	Colistin (*n* = 4)		L-carnitine + Colistin (*n* = 3)		P-value
	mean	SD	mean	SD	
AUC (mg/ml/min)	2.36	0.32	1.93	0.39	0.86
C_max_ (mg/ml)	0.018	0.008	0.014	0.008	0.86

One time *i.p*. injection with 20 mg/kg colistin with or without co-injection of *i.p.* 30 mg/kg L-carnitine.

AUC area under the curve, C_max_ peak plasma concentration.

P-values were calculated by Mann–Whitney U test.

### Colistin-induced nephrotoxicity in mice co-injected with L-carnitine

**Figure 4:**
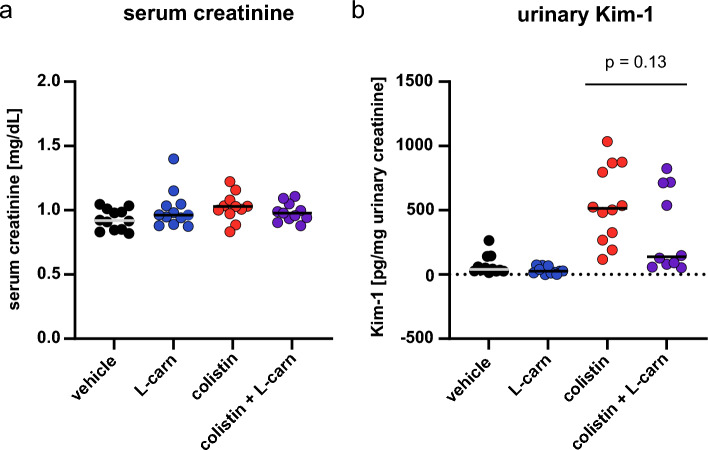
Markers of kidney function and tubular injury. Serum creatinine (**A**) and urinary Kim-1 (B) after daily *i.p*. injection with 20 mg/kg colistin with or without co-injection of 30 mg/kg L-carnitine, for 7 consecutive days. Serum creatinine and urinary Kim-1 were assessed in the samples obtained at or immediately prior to euthanization (day 7). Kim-1 values were normalized to the urinary creatinine values. Experiment was performed 3 times independently. Each data point represents one mouse. P-values were calculated from one-way analysis of variance followed by Tukey’s post-hoc test.

**Figure 5:**
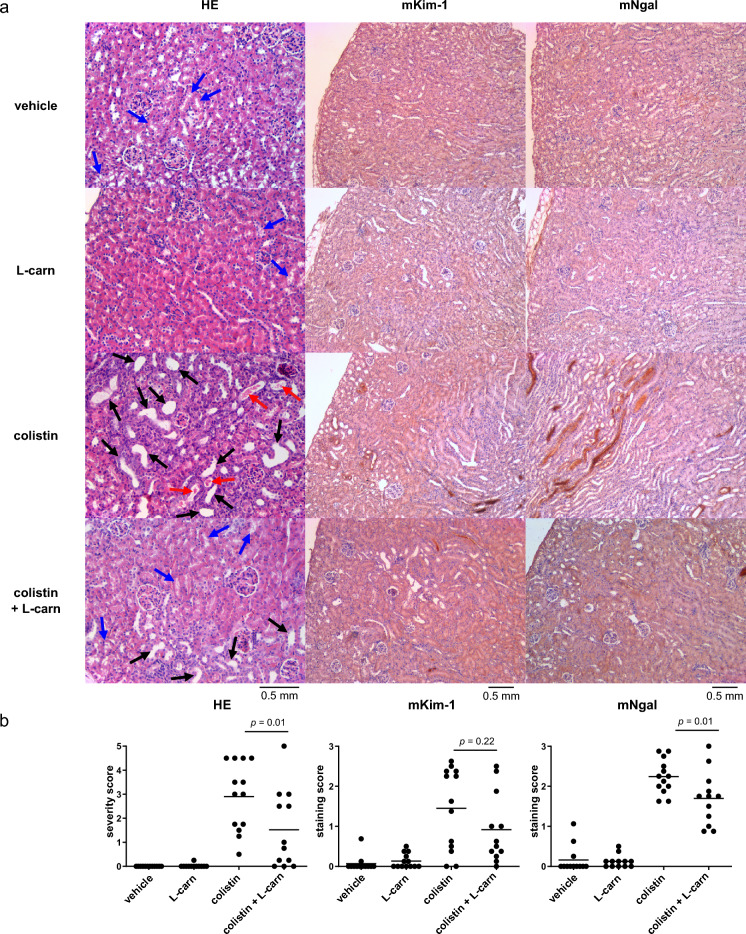
Histological signs of acute kidney injury. (**A**) Hematoxylin and eosin Y (HE) staining, Kim-1, and Ngal stainings of mouse kidney sections after daily *i.p*. injection with 20 mg/kg colistin with or without co-injection of 30 mg/kg L-carnitine, for 7 consecutive days. Brown areas indicate a positive staining. Black arrows: tubular vacuolization and loss of brush border membrane in proximal tubules. Red arrows: tubular protein casts. 20x (HE) and 10 × magnification, scale bar is 0.5 mm. (**B**) Quantification: Severity scores were assigned on a scale of 0–5 in HE-stained kidney sections based on (1) the presence and amount of protein casts, (2) presence and percent area with tubular dilation and (3) loss and area lacking clear brush border membrane structure. Histology staining scores of kidney sections stained for Kim-1 or Ngal were assigned on a scale of 0 (no staining), 1 (mild), 2 (moderate), and 3 (intense), based on the intensity and area stained per section. Each data point represents one mouse. P-values were calculated from one-way analysis of variance followed by Tukey’s post-hoc test.

The nephroprotective effect of L-carnitine was assessed in mice treated with 20 mg/kg of colistin with or without co-injection of 30 mg/kg L-carnitine. The dose of colistin was chosen based on a preliminary experiment to determine the dose sufficient to lead to histological damage in all treated mice. Based on a previous work, 30 mg/kg was considered the dose sufficient to achieve a C_max_ comparable to the L-carnitine concentration used in vitro (1 mM)^41^. In line with our previous study, no differences in serum creatinine were found among groups (Fig. 4A). Thus, assuming comparable glomerular filtration rate among groups, urinary creatinine was used as a normalization element for urinary Kim-1 values. In a previous study, we showed that urinary Kim-1 outperformed urinary Cystatin-C and urinary glucose in the detection of colistin-induced kidney injury in mice^38^. Urinary Kim-1 was significantly elevated in mice treated with colistin. Notably L-carnitine co-injection did not lower the urinary Kim-1 level as compared to the colistin group (Fig. 4B). In histology, all kidney tissues from the mice treated with colistin showed elevated expression of the AKI markers Ngal and Kim-1, tubule dilation, loss of the brush border membranes in the proximal tubules, and the formation of protein casts throughout the kidney tissue, indicating vast tubular damage (Fig. 5A). All these signs of tubular damage were significantly less prevalent in the kidneys from the mice co-injected with L-carnitine (Fig. 5B). When considering histological severity score as the relevant evaluation criteria for AKI (gold standard), the relative risk of colistin-induced AKI was significantly reduced by 50% by L-carnitine co-injection (Table 3).

**Table 3:** Relative risk (RR) of colistin-induced kidney injury confidence intervals.

	Colistin vs L-carnitine + colistin				
Matrix	Criteria	Relative risk	95% CI	z statistic	P-value
Tissue	HE score	0.45	0.23–0.89	2.267	0.02
	Kim-1 score	0.48	0.20–1.16	1.631	0.10
	Ngal score	0.83	0.65–1.07	1.412	0.16
Urine	Kim-1 level	0.47	0.17–1.23	1.448	0.14

*i.p*. injection with 20 mg/kg colistin with or without co-injection of *i.p.* 30 mg/kg L-carnitine, for 7 consecutive days. RR was calculated from the formula: (IE/(IE + IN))/(CE/(CE + CN)), IE: Events of kidney injury in the intervention group (L-carnitine + Colistin), IN: Non-Events of kidney injury in the intervention group (L-carnitine + Colistin), CE: Events of kidney injury in the control group (Colistin), CN: Non-Events of kidney injury in the intervention group (Colistin).

The P-value was calculated according to Sheskin, 2004 (p. 542) using the MedCalc relative risk calculator. A standard normal deviate (z-value) was calculated as ln(RR)/SE(ln(RR)), and the P-value is the area of the normal distribution that falls outside ± z. False positive cut-offs for each criterion category was set to the maximum score or highest value observed in any animal of the vehicle control group across all experiments.

## Discussion

Colistin is a cyclic heptapeptide with an acylated tripeptide side chain that exerts its antimicrobial activity by disrupting the integrity of the bacterial membrane and increasing its permeability, leading to leakage of the cell content, membrane depolarization and cell death^1^. Experiments with chemically modified polymyxin B indicate that the fatty acyl diaminobutyric acid side chain is required for bacterial membrane depolarization and antimicrobial activity^42^. It was previously shown that mitochondria isolated from the central nervous system of mice treated daily with 15 mg/kg *i.v.* colistin for 7 days were characterized by increased mitochondrial permeability transition and mitochondrial membrane depolarization^21^. The permeability transition represents a sudden leakage of solutes across the inner mitochondrial membrane upon opening of the mPTP, a “fail open valve” that releases Ca^2+^ in excess from the matrix. Permanent opening of the mPTP results in mitochondrial depolarization, inhibition of respiration, cessation of ATP synthesis, mobilization of cytochrome C, and apoptosis^43^. In this study, we show that isolated mitochondria exposed to colistin undergo rapid depolarization of the mitochondrial inner membrane, indicating that mitochondrial damage is likely to be an early event in colistin-induced kidney injury. This reconciles with the well-characterized depolarizing effect exerted by fatty acids on the mitochondrial inner membrane, irrespective of their chemical structure. The nature of fatty acid-induced depolarization seems to be pleiotropic, encompassing destabilization of the mitochondrial inner membrane, mitochondrial uncoupling, and opening of the mPTP^44–48^. Moreover, we found that colistin-induced depolarization is sensitive to cyclosporin A, a potent inhibitor of mitochondrial cyclophilin D (CyPD), one of the components of the mPTP, whose expression and function are required for the onset of the permeability transition^49^. In a similar vein, co-incubation with L-carnitine, a quaternary amine that desensitizes the mPTP by reducing the transmembrane electrical potential, prevents the permeability transition of mitochondria exposed to colistin^48^. Taken together, our results suggest that colistin-induced mitochondrial depolarization occurs primarily upon opening of the mPTP.

We found that primary cultured mouse tubular cells exposed for 24 h to colistin at an extracellular concentration of 100 µM indeed displayed mitochondrial and ER damage, and that both were prevented by co-incubation with 1 mM L-carnitine. Under physiological conditions, most Ca^2+^ is stored in the ER lumen. The Ca^2+^ released from the ER into the cytoplasm during signalling is rapidly reabsorbed in the ER lumen, with only a small fraction entering the mitochondria. Conversely, in conditions of ER stress, Ca^2+^ is released in excess and accumulates in the mitochondria, causing the mPTP opening and the depolarization of the inner membrane^50^. Meanwhile, mitochondria are an important source of reactive oxygen species (ROS), whose production not only drives mitochondrial dysfunction but also contributes to the redox signalling to the other organelles, including the ER^51^. High levels of ROS facilitate Ca^2+^ release from the ER and accumulation into the mitochondria, triggering the permeability transition^52^. Previous studies in rodents have shown that colistin-induced apoptosis involves both the mitochondrial (e.g., downregulation of Bcl-2 and upregulation of cytochrome C and Bax) and the ER pathways (upregulation of Grp78)^20,21^. Moreover, experiments in rat renal tubular (NRK-52E) cells showed the accumulation of fluorescently labelled polymyxins in both the ER and mitochondrial compartments^53^. A strong positive correlation exists between mitochondrial membrane potential and ROS production, whereby hyperpolarization drives the production of ROS^51,52,54^. Thus, depolarizing agents such as colistin should not increase ROS production. We lean towards the conclusion that in cells, colistin-induced permeability transition might occur because of the direct interaction with the mitochondria as well as upon prolonged release of Ca^2+^ from the ER lumen. This is also suggested by the overexpression of the chaperon GRP78/BiP, which appears to play an important role in the mobilization of Ca^2+^ from the ER to the mitochondria^50,55^. Because colistin is a substrate of the carnitine/organic cation transporter 2 (OCTN2, *SLC22A5*), we also considered the possibility that co-incubation with L-carnitine reduced colistin intracellular accumulation and toxicity. However, a substantial inhibition of OCTN2-mediated uptake of colistin in the presence of L-carnitine is unlikely at the concentrations used in our experiments due to the nature of their binding within the OCTN2 pocket^15^. A drug-drug interaction in vivo is considered likely when the ratio between the C_max_ measured in plasma and the inhibitory constant (K_i_) measured in vitro is higher than 0.1. Based upon the colistin C_max_ achieved in our experiments (10–20 μM) and the K_i_ for carnitine transport calculated in our previous work (∼22 mM)^15^, the calculated C_max_/K_i_ ratio is approximately 0.001. This is substantiated by the pharmacokinetic data showing that L-carnitine co-injection did not alter the plasma concentration of colistin.

L-carnitine co-incubation fully rescued colistin-induced damage in isolated mitochondria as well as in primary cultured tubular cells, whereas the protection in vivo was only partial. One explanation is that the doses and/or dosing regimens used in this study were suboptimal. It has been shown that the optimal dose of cyclosporin A for in vivo desensitization of the mPTP is 5 mg/kg of body weight, with lower and higher doses being less effective^56^. Our experiments in isolated mitochondria and in tubular cells indicated that 1 mM L-carnitine efficiently desensitizes the opening of the mPTP and prevents colistin-induced toxicity. The bulk of L-carnitine pharmacokinetic studies focused on *i.v.* infusions in humans. In one study, after 30 mg of L-carnitine per kg of body weight was administered intravenously to healthy adult males, the average plasma C_max_ was 1.5 mM^57^. In another study, the mean C_max_ after receiving 20 mg of L-carnitine/kg *i.v.* was approximately 1 mM^41^. Extrapolating from these studies, we believe that the L-carnitine C_max_ achieved in mice upon *i.p.* injection of 30 mg of L-carnitine/kg body weight may not be far from the L-carnitine concentration effective in vitro. In all these studies, the plasma concentration of L-carnitine returned to within the normal range (approximately 50 µM) within 2–4 h, suggestive of an extensive urinary loss of L-carnitine, arguably as a result of the saturation of the main renal L-carnitine reabsorption system OCTN2 (K_m_ = 5–15 µM)^17^. The rapid renal clearance of L-carnitine observed in humans may also apply to our animal experiments. It has been previously shown that cyclosporin A could protect from tumor necrosis factor-alpha (TNF-alpha)-induced acute hepatic injury when administered as multiple-interval injections but not as pre- or concomitant single-injections^56,58^. This may indicate that to obtain a prolonged and effective desensitization of the mPTP in vivo, a steady-state plasma elevation of the desensitizer agent must be achieved.

Several in vitro and in vivo studies have shown that antioxidants have a protective effect during treatment with colistin^59^. However, in most cases the translation to the clinical setting is hampered by the lack of safety data in humans for the respective protective agents. Conversely, clinical studies and anecdotal evidence indicate that L-carnitine is safe at doses comparable to those used in this study. We have learned that L-carnitine can be safely administered repeatedly at doses as high as 100 mg/kg as an effective antidote for valproic acid overdose^60^. We believe that a similar dosing scheme might be safely applied during treatment with colistin to reduce the risk and/or severity of acute kidney injury.

### Supplementary Information

Supplementary Information.

## Data Availability

All data generated or analyzed during this study are included in this published article and in the supplementary files.
