# Supplementary material for: L-carnitine co-administration prevents colistin-induced mitochondrial permeability transition and reduces the risk of acute kidney injury in mice
Source: Sci Rep. 2024 Jul 16;14:16444. doi: 10.1038/s41598-024-67171-x (PMC11252255; doi:10.1038/s41598-024-67171-x)
Supplement: Supplementary file 1 — Supplementary Information. [file 41598_2024_67171_MOESM1_ESM.docx]

**L-carnitine Prevents Colistin-induced Mitochondrial Permeability Transition and Reduces the Risk of Acute Kidney Injury in Mice**

Sophia L. Samodelov^1^, Zhibo Gai^1,2^, Francesca De Luca^1^, Klara Haldimann^3^, Sven N. Hobbie^3^, Daniel Müller^4^, Gerd A. Kullak-Ublick^1,5^, Michele Visentin^1^

**Supplementary Information**

**Oxygen consumption rate (OCR) data analysis and interpretation**

ΔOCR was calculated as the fractional difference ((value – baseline)/baseline), in the presence or absence of L-carnitine and normalized against the same volume of water injected as colistin for each titration (volumes differed between 1μm and 10 μM colistin) reported in the body of the manuscript (Figure 1E). Baseline values used in the calculation of the fractional difference was the steady state OCR after succinate or L-carnitine addition for colistin-only or colistin + L-carnitine-treated samples, respectively. The intention of displaying the data in this way was to focus the reader’s attention to the effect of L-carnitine on the changes in oxygen consumption observed under colistin treatment. However, the addition of L-carnitine alone affects OCR, as shown below in Fig. S1. Similar to treatment with Cyclosporin A (CsA), L-carnitine leads to a reduction in OCR, arguably because of slight hyperpolarization.

**Figure S1. Additional data on mitochondrial membrane potential and oxygen consumption rate** **with ΔOCR calculated to succinate steady state**. The effect of 250 µM L-carnitine, 1 µM CsA, and of 1 and 10 µM colistin, in presence and absence of L-carnitine or CsA, on OCR of isolated mitochondria detected in the presence of succinate as a substrate, and their respective control values obtained by injecting the same volume of water (shown in black). Control (water) values and treatment values are displayed side by side, separated with dotted lines between conditions. Control values are missing for L-carnitine and CsA due to the setup of the experiment. ΔOCR is expressed as the fractional difference ((value – baseline)/baseline) to succinate steady state (baseline) for all data shown.

**Liquid chromatography and tandem mass spectrometry – colistin quantification in kidney and serum**

**Table S1. LC-MS settings for colistin quantification**

| Time [min] | Loading flow rate [ml/min] | Loading %A | Loading %B | Loading %C | Eluting flow rate [ml/min] | Eluting %A | Eluting %B |
| --- | --- | --- | --- | --- | --- | --- | --- |
| 0.00 | 2.00 | 100 |  |  | 0.50 | 99 | 1 |
| 0.50 | 0.20 | 100 |  |  | 0.45 | 99 | 1 |
| 1.00 | 1.00 |  | 100 |  | 0.50 | 85 | 15 |
| 1.17 | 2.00 |  |  | 100 | 0.50 | 70 | 30 |
| 2.00 | 2.00 |  |  | 100 | 0.50 | 70 | 30 |
| 2.50 | 2.00 | 70 | 30 |  | 0.60 |  | 100 |
| 4.00 | 2.00 | 100 |  |  | 0.50 | 99 | 1 |
| 5.50 | 2.00 | 100 |  |  | 0.50 | 99 | 1 |

Mobile phase A: aqueous formic acid (0.5% v/v)

Mobile phase B: 0.5% (v/v) formic acid in acetonitrile/water 90/10 (v/v)

Mobile phase C: acetonitrile/2-propanol/acetone 1/1/1 (v/v/v)

Loading = loading pump

Eluting = eluting pump

**Mitochondrial membrane potential of control and protective treatment groups against colistin**

**Figure S2. Mitochondrial membrane potential of control and protective treatment groups against colistin.** Mitochondria freshly isolated from mouse kidney were incubated for 1 hour with water (control), 1 µM colistin, 250 µM L-carnitine, or 1 µM CsA, then mixed with Rho123 solution and the decline of the fluorescent signal was monitored over time. Data represent the mean ± SD from six independent experiments and expressed relative to the baseline fluorescent signal (F0) recorded in the absence of mitochondria.
